# Current status and research gaps of neuromodulation in the rehabilitation of knee osteoarthritis: a scoping review

**DOI:** 10.3389/fneur.2026.1845945

**Published:** 2026-07-15

**Authors:** Hengjia Liu, Qiang Tang

**Affiliations:** 1 Heilongjiang University of Chinese Medicine, Harbin, China; 2 The Second Affiliated Hospital of Heilongjiang University of Chinese Medicine, Harbin, China

**Keywords:** electroacupuncture, knee osteoarthritis, neuromodulation, pain, rehabilitation, transcranial direct current stimulation, transcutaneous electrical nerve stimulation

## Abstract

**Background:**

Knee osteoarthritis (KOA) is a highly prevalent degenerative joint disease with a growing global disease burden, yet traditional interventions have limited efficacy. Neuromodulation has emerged as a promising non-pharmacological rehabilitation strategy for KOA, but existing reviews have not systematically synthesized the clinical protocols, efficacy characteristics, and full spectrum of research gaps for mainstream neuromodulation techniques. This scoping review was conducted in accordance with the Preferred Reporting Items for Systematic Reviews and Meta-Analyses Extension for Scoping Reviews (PRISMA-ScR) guidelines, aiming to describe the current application status and identify critical research gaps.

**Methods:**

A systematic search was performed in PubMed and Web of Science databases for clinical studies on neuromodulation for KOA published between May 13, 2021 and May 13, 2026. Two researchers independently completed literature screening, data extraction, and cross-verification according to pre-specified inclusion and exclusion.

**Results:**

A total of 131 records were initially retrieved. After deduplication, title/abstract screening, and full-text evaluation, 23 studies were finally included, comprising 18 randomized controlled trials, 3 self-controlled before-after trials, 1 retrospective case series, and 1 cross-sectional study. The included studies covered three core categories of neuromodulation: peripheral neuromodulation (TENS, NMES, PNS, electroacupuncture, electrical dry needling, tVNS), central neuromodulation (tDCS), and combined neuromodulation (TENS + tDCS). All techniques demonstrated certain efficacy in pain relief and functional improvement, but intervention protocols varied widely, and optimal parameters have not been standardized.

**Conclusion:**

Neuromodulation, as a non-pharmacological rehabilitation strategy, has broad application prospects in KOA treatment. However, existing studies still have deficiencies in intervention standardization, long-term efficacy evaluation, and mechanism exploration. Future high-quality, large-sample studies are needed to optimize clinical protocols and clarify mechanisms of action, providing evidence support for the standardized application of neuromodulation in KOA rehabilitation.

**Systematic review registration:**

Retrospective registration for this study on Open‑Science Framework (OSF). The publicly available link is https://osf.io/vyz8q/overview.

## 1 Introduction

Knee osteoarthritis (KOA) is a degenerative joint disease characterized by articular cartilage degeneration, destruction, hyperplasia, and inflammation. Its typical manifestations include joint pain, swelling, stiffness, crepitus, and limited mobility. It is more prevalent in middle-aged and elderly individuals, with a higher incidence in women, possibly related to altered joint metabolism caused by postmenopausal estrogen decline. The etiology of KOA remains unclear and may be associated with age, obesity, genetics, trauma, and other factors. Studies have shown that the global prevalence of osteoarthritis continues to rise, particularly in rapidly aging countries such as China. KOA not only causes individual functional impairment but also imposes a heavy burden on public health systems (1).

In recent years, in-depth research on KOA pathological mechanisms, biomarkers for disease progression prediction, and pain regulatory pathways has laid a critical theoretical foundation for the development of targeted non-pharmacological therapies. In the field of disease progression and biomarkers, a series of studies by Hunter’s team confirmed that the T2 relaxation time of superficial articular cartilage and its longitudinal changes are significantly correlated with radiographic joint space width (JSW) loss and sustained elevation of WOMAC pain scores, making it an effective predictive biomarker for KOA progression (2).

In the field of KOA risk factors and pain mechanisms, Neogi’s team, based on large-sample epidemiological studies, identified obesity and knee injury as the core modifiable risk factors for KOA onset and progression, laying the foundation for the development of KOA primary prevention strategies (3, 4). Meanwhile, relying on the Multicenter Osteoarthritis Study (MOST), the team systematically evaluated the correlation between somatosensory assessments such as pressure pain threshold (PPT) and knee pain in KOA patients, providing methodological references for quantitative assessment of chronic pain (5, 6).

Arendt-Nielsen’s team focused on neuropathic pain mechanisms, quantitative sensory testing (QST), and central sensitization assessment in KOA, confirming that KOA patients exhibit extensive peripheral and central sensitization, with the degree of sensitization positively correlated with disease severity (7, 8). The team also developed a simplified bedside QST kit (QuantiPain) for evaluating pain facilitation and inhibition mechanisms in KOA patients, and verified its test–retest reliability and validity (9).

The International Neuromodulation Society (INS) defines neuromodulation as: “Neuromodulation is a technology that acts directly on nerves. It alters or regulates neural activity by delivering electricity or drugs directly to the target area.” (Website: www.neuromodulation.com/about_neuromodulation) “neuromodulation” is used in a broad clinical sense in this review, covering interventions that act on peripheral or central neural regulatory pathways. In the field of KOA rehabilitation, such neuromodulation interventions mainly include: peripheral neuromodulation, “neuromodulation” is used in a broad clinical sense in this review, covering interventions that act on peripheral or central neural regulatory pathways—transcutaneous electrical nerve stimulation (TENS), peripheral nerve stimulation (PNS), neuromuscular electrical stimulation (NMES), interferential current stimulation (IFC), peripheral nerve block (PNB), lumbar sympathetic block (LSB), electroacupuncture (EA), electrical dry needling (EDN); central neuromodulation - transcranial direct current stimulation (tDCS), repetitive transcranial magnetic stimulation (rTMS), spinal cord stimulation (SCS), vagus nerve stimulation (VNS), transcutaneous vagus nerve stimulation (tVNS). These methods are important non-pharmacological treatment modalities with advantages of simplicity, high efficiency, and minimal adverse reactions, providing new insights for clinical management of KOA.

Although the number of clinical studies on neuromodulation for KOA has been increasing annually, no unified consensus has been reached on the optimal protocols, efficacy differences, and underlying mechanisms of various techniques. Therefore, this scoping review summarizes clinical evidence from the past 5 years to provide a reference for optimizing neuromodulation rehabilitation protocols for KOA.

## 2 Methods

### 2.1 Search scope

Studies published in PubMed and Web of Science databases over the past 5 years (May 13, 2021–May 13, 2026) were retrieved.

### 2.2 Inclusion criteria

1. Study subjects: Adult patients (≥18 years old) diagnosed with knee osteoarthritis (KOA, including patellofemoral osteoarthritis);
2. Intervention measures: Primary intervention is any neuromodulation technique (EA, EDN, tDCS, rTMS, TENS, PNS, VNS, tVNS, NMES, IFC, SCS, PNB, LSB) or a combination of two or more neuromodulation techniques;
3. Study types: Original human clinical studies (randomized controlled trials, non-randomized controlled trials, self-controlled before-after trials, etc.).

### 2.3 Exclusion criteria

1. Non-clinical studies: Animal experiments, literature reviews, systematic reviews, meta-analyses, guidelines, case reports, letters, surveys;
2. Ineligible study subjects: Patients with knee pain or dysfunction after total knee arthroplasty, patients with only knee pain but no confirmed KOA diagnosis;
3. Ineligible intervention measures: Neuromodulation only as adjuvant therapy (e.g., <1 time per week), or treatments dominated by drugs/surgery/conventional acupuncture/dry needling/tuina/moxibustion/nursing; combined use of neuromodulation with other therapies;
4. Missing data: Study protocols only, retracted/unavailable full texts, studies with unclear intervention protocols.

### 2.4 Search strategy

Search formula: [((((knee osteoarthritis OR knee OA) AND (electroacupuncture OR electrical dry needling OR Transcranial Direct Current Stimulation OR Repetitive Transcranial Magnetic Stimulation OR Transcutaneous Electrical Nerve Stimulation OR Peripheral Nerve Stimulation OR Vagus Nerve Stimulation OR Transcutaneous Vagus Nerve Stimulation OR Neuromuscular Electrical Stimulation OR interferential current stimulation OR spinal cord stimulation OR Peripheral Nerve Block OR lumbar sympathetic block OR neurolysis) AND (Humans OR Clinical Study OR Clinical Trial)) NOT (meta)) NOT (protocol))] NOT (review).

### 2.5 Literature processing

All retrieved literature was imported into Zotero. Two researchers independently performed literature screening, data extraction, and cross-verification. Any discrepancies were resolved through consensus between the two authors, with no third-party involvement. The PRISMA flow diagram was generated using RevMan 5.4.

### 2.6 Data extraction

Two researchers extracted core data from eligible studies, including author, publication year, country, study type, sample size, neuromodulation type, specific protocol (acupoints/parameters/frequency/treatment duration), control measures, and efficacy indicators. Cross-verification was performed after extraction to ensure data accuracy. Qualitative description was adopted, with intervention protocols and efficacy indicators classified and summarized to extract methodological details.

## 3 Results

### 3.1 Literature screening results

#### 3.1.1 Process

**Figure 1:**
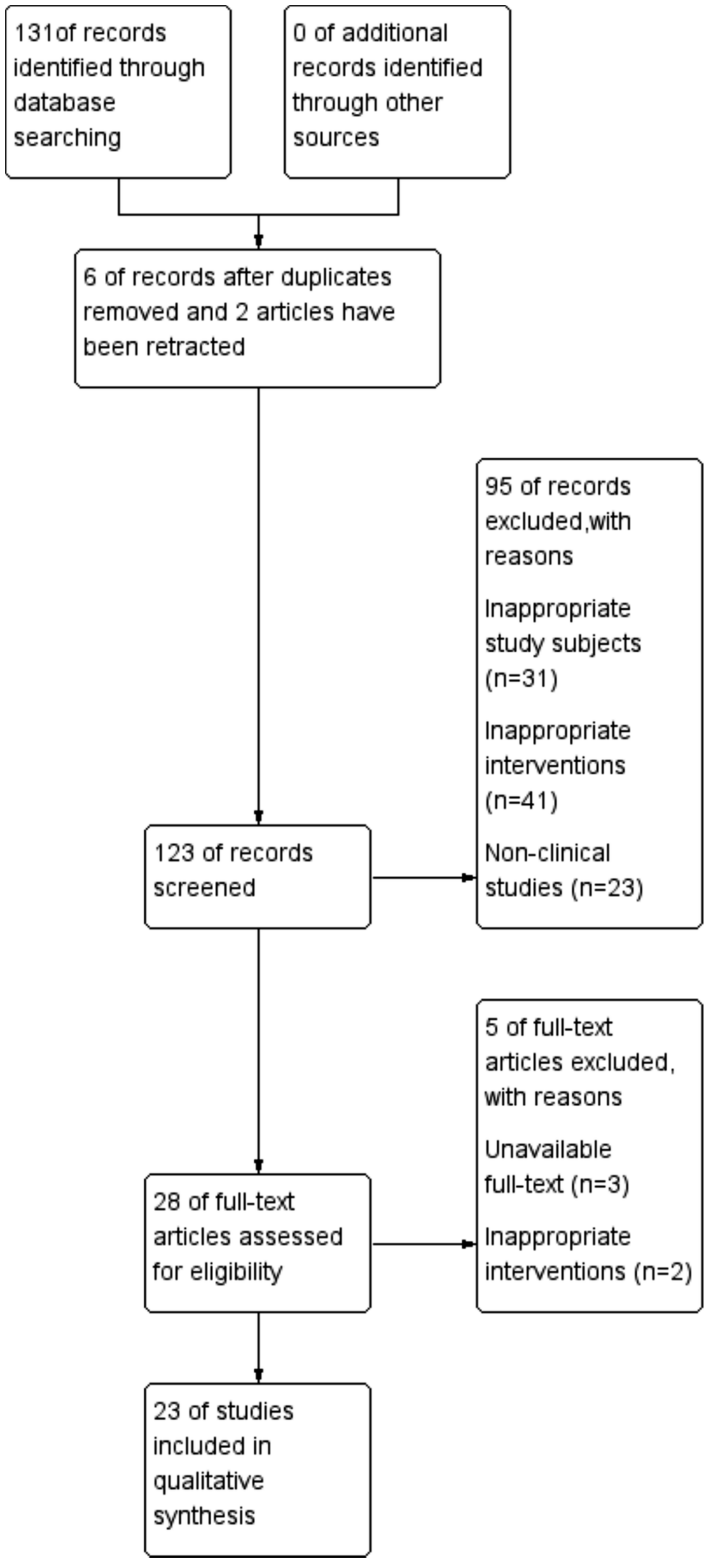
PRISMA flow diagram.

A total of 131 literatures were initially retrieved (117 from PubMed, 14 from Web of Science). After deduplication, retraction check, and manual verification in Zotero, 123 literatures remained; 28 literatures were retained after title and abstract screening, and 23 literatures were finally included after full-text evaluation. The PRISMA flow diagram is shown in Figure 1.

#### 3.1.2 Characteristics of Included Studies

The characteristics of included studies are summarized in Table 1.

**Table 1:** Characteristics of included studies.

Author	year	country	study type	Sample size (experimental/control)	Neuromodulation type	Specific protocol	Control measures	Efficacy indicators
Jean Carlos Constantino Silva (34)	2026	Brazil	Double-blind, randomized, controlled feasibility clinical trial (RCT)	8/9/8	Neuromuscular Electrical Stimulation (NMES, including two current modes: AUSSIE medium-frequency alternating current, Functional Electrical Stimulation (FES) pulse current)	Electrode placement: 6 pairs of 50 × 50 mm self-adhesive electrodes attached to the motor points of the vastus medialis and vastus lateralis muscles of the dominant and contralateral lower limbs with knee OA Stimulation parameters: AUSSIE current: carrier frequency 1 kHz, burst frequency 50 Hz, on–off ratio 10s:10s, pulse width 200 μs, current intensity adjusted to the subject’s maximum sensory tolerance level (average 46.6mA) FES current: burst frequency 50 Hz, on–off ratio 10s:10s, pulse width 200 μs, current intensity adjusted to the subject’s maximum sensory tolerance level (average 82.32mA) Frequency: 3 times per week Treatment course: 4 consecutive weeks, total 12 treatments, single treatment duration 20 min	No-intervention blank control group (no electrical stimulation intervention, only completed baseline and outcome assessments)	Primary feasibility indicators: recruitment rate, treatment adherence, subject satisfaction, safety (adverse event rate) Secondary clinical efficacy indicators: Visual Analogue Scale (VAS) pain score, Timed Up and Go (TUG) test, 6-meter walk test
Vinod Dasa (13)	2022	United States	Extension study of a multicenter, randomized, double-blind, sham-controlled clinical trial (14-week extension follow-up based on the 12-week parent study)	40/22	NMES	Electrode placement: Vastus medialis obliquus, rectus femoris (core quadriceps muscles), fixed with a knee conductive garment with built-in electrodes Stimulation parameters: Active NMES group used CyMedica NMES system, output voltage 0.5–16.4 V (adjusted according to impedance, intensity 0–100 levels adjustable by patients); Sham NMES group maximum output intensity only level 5, maximum voltage 3.18 V Frequency: 2 times daily, 5 days per week (weekdays) Treatment course: 14 weeks in the extension study phase, total 26 weeks (including 12 weeks of the parent study), single treatment duration 20 min, required cumulative treatment duration ≥2,800 min	Low-voltage sham NMES stimulation, identical electrode placement, treatment frequency, and course to the active stimulation group, only limited maximum output voltage, no effective muscle contraction stimulation	Core efficacy indicators: VAS specified activity pain score, patient response rate of VAS pain relief ≥50% Secondary efficacy indicators: Western Ontario and McMaster Universities Osteoarthritis Index (WOMAC) pain/stiffness/function subscales, isometric quadriceps strength, patient global impression of change, treatment satisfaction, adverse event rate
Camila Cadena De Almeida (35)	2025	Brazil	Multicenter, randomized, double-blind, placebo-controlled, parallel-group four-arm clinical trial (RCT)	25/25/25/25	NMES	Electrode placement: 2 channels of 25 cm^2^ self-adhesive electrodes attached around the patella, channel 1 near the medial femoral condyle and fibular head, channel 2 near the lateral femoral condyle and medial tibial plateau, only stimulating the most symptomatic knee Stimulation parameters: TENS group: pulse frequency 100 Hz, phase duration 200 μs IFC group: burst frequency 100 Hz, carrier frequency 4,000 Hz (burst duration 10 ms, duty cycle interval 10 ms), phase duration 125 μs AUSSIE group: burst frequency 100 Hz, carrier frequency 4,000 Hz (burst duration 4 ms, duty cycle interval 16 ms), phase duration 125 μs Single stimulation duration 20 min for all groups, current intensity gradually increased to the subject’s maximum tolerable sensory threshold every 5 min Frequency: 3 times per week Treatment course: 4 consecutive weeks, total 12 treatments	Placebo NMES stimulation, identical electrode placement, treatment frequency, course, and single duration to the active stimulation group; only 100 Hz, 200 μs electrical stimulation was given for the first 40 s, then the current intensity gradually decreased to 0, no effective electrical stimulation output throughout the treatment, only keeping the device on and electrodes connected to achieve blinding	Primary efficacy indicators: Visual Analogue Scale (VAS) pain score (0–10 points), Western Ontario and McMaster Universities Osteoarthritis Index (WOMAC, 0–96 points, including pain/stiffness/function subscales), Pressure Pain Threshold (PPT) Secondary efficacy indicators: 6-min walk test (aerobic capacity), gait speed, lower limb muscle strength, functional activity ability, knee surface temperature, postural stability, fall risk
Wenrui Jia (17)	2025	China	Prospective clinical intervention study (self-controlled before and after, small sample pilot)	3	Electroacupuncture (EA)	Acupoints: mandatory points (Dubi ST35, Neixiyan EX-LE5, Ququan LR8, Xiyangguan GB33, Ashi points); auxiliary points (selected along the stomach/gallbladder/spleen meridians according to the pain location) Parameters: 2/100 Hz dense-disperse wave, current intensity adjusted to slight tremor of the needle body Frequency: 1–2 times per week, total 24 times in 8 weeks Duration: 30 min per session	None (only self-controlled before and after intervention, no blank/sham control group)	Clinical indicators: WOMAC osteoarthritis index, Numerical Rating Scale (NRS) pain score Serum indicators: pro-inflammatory factors (IL-8, IL-18, IL-1β, TNF-*α*, MCP-1), anti-inflammatory factors (IL-10, IL-13, IL-4), matrix metalloproteinases (MMP-3, MMP-13) Immune indicators: peripheral blood T cell receptor (TCR) diversity (D50, diversity index DI, Shannon entropy) Transcriptome indicators: neutrophil mRNA (CXCL2, IRF8, PEAR1, SMPD3)
Tian-Qi Wang (15)	2021	China	Multicenter, randomized, double-blind, sham-acupuncture controlled clinical trial (RCT), with a healthy volunteer blank control group included	30/30/30	Electroacupuncture (EA)	Acupoints: main points (mandatory) are Dubi (ST35), Neixiyan (EX-LE5), Ququan (LR8), Xiyangguan (GB33), Ashi points; auxiliary points selected according to syndrome differentiation based on pain location, electrodes fixed to the needle handles of LR8, GB33 and 2 auxiliary points Stimulation parameters: HANS-200A acupoint nerve stimulator was used, waveform 2/100 Hz, current intensity adjusted to slight vibration of the needle body, single electrical stimulation duration 30 min Frequency: 3 times per week Treatment course: 8 consecutive weeks, total 24 treatments	Sham acupuncture (SA) control group: shallow needling at 8 non-acupoint sites, no electrical stimulation, no needle manipulation to achieve deqi, identical treatment frequency, course, and single duration to the electroacupuncture group Healthy volunteer blank control group: no acupuncture/electrical stimulation intervention, only completed baseline sample collection and assessments	Clinical efficacy indicators: total score and pain/stiffness/function subscales of Western Ontario and McMaster Universities Osteoarthritis Index (WOMAC), Numerical Rating Scale (NRS), SF-12 Health Survey (physical/mental total scores), clinical response rate (proportion of patients with WOMAC total score decreased by ≥50% from baseline) Biological mechanism indicators: fecal intestinal flora 16S rRNA sequencing (flora diversity, species composition, abundance of differential genera)
Yong LIU (16)	2022	China	Single-center, randomized, controlled clinical trial (RCT), double-blind design for outcome assessors and statisticians	30/30/30	Electroacupuncture (EA)	Acupoints: basic acupoints are Liangqiu (ST34), Xuehai (SP10), Dubi (ST35), Neixiyan (EX-LE4), Yanglingquan (GB34), Heding (EX-LE2), Sanyinjiao (SP6), needling on unilateral/bilateral affected side, electrical stimulation implemented based on the above acupuncture points Stimulation parameters: low-frequency pulse current, continuous wave, single electrical stimulation duration 30 min Frequency: once daily, rest 1 day after every 6 consecutive days of treatment Treatment course: total 21 days (3 weeks)	Ordinary acupuncture group: acupuncture at the same acupoints, no electrical stimulation, needle retention for 30 min, identical treatment frequency and course to the electroacupuncture group Drug control group: oral celecoxib capsules (0.2 g per dose, once daily) for 21 consecutive days	Core clinical efficacy indicators: Western Ontario and McMaster Universities Osteoarthritis Index (WOMAC), Visual Analogue Scale (VAS) pain score, total clinical effective rate Biological mechanism indicators: serum pro-inflammatory factors (IL-1β, TNF-*α*) levels
Aaron Mates (14)	2026	USA	Retrospective case series study	4 patients with chronic pain from Kellgren-Lawrence grade 4 severe knee osteoarthritis	Peripheral Nerve Stimulation (PNS)	Stimulation target: infrapatellar saphenous nerve (IPS) Device: Freedom® PNS System (Curonix LLC), using high-frequency electromagnetic coupling (HF-EMC) technology, implanted 4/8-contact electrode array + independent receiver, paired with external wearable transmitter components Stimulation parameters: subthreshold stimulation, fixed frequency 1,499 Hz, current intensity adjustable according to subject tolerance Treatment course: first complete diagnostic nerve block + PNS trial, implant permanent stimulation system after successful trial (pain relief >50%), long-term wear and use	No concurrent parallel control group, self-controlled before and after (baseline level, post-trial, long-term follow-up)	Primary indicators: pain response rate (proportion of patients with pain relief >50%), Verbal Rating Scale (VRS, 0–10 points, assessing pain intensity) Secondary indicators: long-term pain relief rate, physical function improvement, quality of life, analgesic drug usage, adverse event rate
James Dunning (18)	2025	Multicenter, main study site in the United States	Multicenter, single-blind, parallel-group randomized clinical trial (RCT)	195/197/194	Periosteal and intra-articular electrical dry needling	Stimulation sites: standardized 23-point protocol for the knee joint (4 intra-articular points, 8 periosteal points, 11 points around the joint line) Stimulation parameters: low frequency 2 Hz, pulse width 250 μS, biphasic continuous wave, maximum tolerable intensity Intervention frequency: initial 6 weeks, 1–2 times per week (total 8–10 times); subsequent 6 months: booster treatment every 4 weeks/8 weeks or no booster Treatment duration: 30 min per session, total intervention period 30 weeks	No booster treatment group (blank control)	Primary indicator: WOMAC osteoarthritis index total score Secondary indicators: Numerical Pain Rating Scale (NPRS), WOMAC pain/stiffness/function subscales, analgesic drug dosage, Global Rating of Change (GROC) score, adverse event rate
IRFAN ULLAH (11)	2026	United Kingdom	Single-arm, prospective home usability pre-experiment (preliminary feasibility assessment)	11	TENS	Site: knee joint (4 50 mm × 50 mm fabric electrodes precisely fitted around the knee) Parameters: biphasic pulse, stimulation voltage 14–130 V, adjustable intensity/duration Frequency: dual-mode stimulation (pulse frequency not specified) Treatment course: 7 consecutive days of home use, once daily for 30 min, additional 30-min session allowed; electrodes can be used after moistening with water, machine washable and reusable	None (single-arm design)	Pain: Visual Analogue Scale (VAS), pain relief rate Usability: device ease of use, wearing comfort, electrode adhesion stability, learning cost Safety: skin irritation, device tolerance, electrode impedance changes Function: knee joint mobility improvement
S. Reichenbach (36)	2022	Switzerland	Multicenter, parallel, 1:1 randomized, double-blind, placebo-controlled clinical trial (RCT)	108/112	TENS	Stimulation site: medial and lateral joint lines of the affected knee (electrodes placed perpendicular to the limb) Stimulation parameters: individualized low-frequency, high-frequency or pulsed TENS; individualized intensity Intervention frequency: 4 times in week 1, 3 times in week 2, 2 times in week 3 Treatment duration: ≤60 min per session, total course 3 weeks	Placebo TENS (automatically slowly powers off after 45 s of electrification, device display remains normal)	Primary indicator: WOMAC pain scale score after 3 weeks Secondary indicators: WOMAC function/stiffness/total score, VAS pain score, Hospital Anxiety and Depression Scale, participation score, analgesic drug usage, adverse event rate, dropout rate, pain response rate (30%/50% relief)
Yu Xu (10)	2025	China	Randomized, double-blind, controlled clinical trial	40/40 (38/37 completed follow-up finally)	TENS	Site: experimental group (knee high nerve density areas: quadriceps tendon, patellar ligament, medial joint line, superomedial knee area); control group (traditional knee pain areas) Parameters: frequency 60–100 Hz, pulse width 100–250 μs, biphasic pulse, individualized low-frequency/high-frequency/burst TENS Treatment course: 5 times per week, ≤30 min per session, total 2 weeks	Electrodes attached to traditional knee pain areas, identical TENS parameters and course to the study group	VAS pain score, WOMAC scale (pain/stiffness/function/total score), adverse events (local skin reactions)
Emmanuel Maheu (12)	2022	France	Multicenter, prospective, randomized, single-blind RCT	55/55	Wearable Transcutaneous Electrical Nerve Stimulation (W-TENS)	Site: high-frequency channel (100 Hz): attached to the medial knee saphenous nerve area (2 pieces of 50 × 50 mm) low-frequency channel (2 Hz): attached to the quadriceps muscle (2 pieces of 50 × 90 mm) Parameters: high frequency (100 Hz) produces tingling sensation, low frequency (2 Hz) produces light muscle contraction, intensity patient-controlled Treatment course: 2 times daily, 5 days per week, for 3 months; can be extended for another 3 months	Weak opioids (tramadol, dihydrocodeine, etc.), used as needed	Primary indicators: 3-month pain intensity (NRS), treatment-related adverse events (TRAEs) Secondary indicators: WOMAC (pain/function/stiffness), EQ-5D, PGIC, response rate (≥30%/≥50% pain relief), safety
Gehad Gamal Elsehrawy (19)	2025	Egypt	Single-blind, sham-controlled, randomized clinical trial (RCT)	34/34	tVNS	Stimulation site: active group left cymba concha; sham group earlobe Stimulation parameters: 25 Hz, pulse width 250 μS, current 0.25–2.0 mA, tolerable tingling sensation Intervention frequency: 30 min daily, 3 times per week, for 12 weeks Treatment duration: 12 weeks of intervention, follow-up to 4 weeks after completion	Sham tVNS (electrode attached to earlobe, current automatically attenuates after 45 s)	VAS pain score, Pressure Pain Threshold (PPT), Central Sensitization Inventory (CSI), KOOS score, Timed Up and Go test, Chair Stand test, PD-Q, DN4, Hospital Anxiety and Depression Scale (HADS), adverse event rate
Kosaku Aoyagi (20)	2025	USA	Single-arm pilot clinical trial (Pilot trial)	30	tVNS	Stimulation site: cymba concha (exclusive innervation area of the auricular branch of the vagus nerve) Stimulation parameters: intensity ≤15 mA, frequency 25 Hz, pulse width 250 μS, 30-s on/off cycle Intervention frequency: single session Treatment duration: 60 min per session	No control group (single-arm design)	Knee pain: 0–10 Numerical Rating Scale (NRS) during 20-meter walking, minimum clinically important improvement rate (≥1.5/10) Central pain mechanism: Pressure Pain Threshold (PPT), Temporal Summation (TS) of mechanical pain, Conditioned Pain Modulation (CPM) Parasympathetic function: high-frequency power of heart rate variability (HF, 0.15–0.40 Hz) Safety/feasibility: intervention completion rate, adverse event rate, subject acceptance
Chiyoung Lee (37)	2025	USA	Double-blind, randomized, sham-controlled, parallel-group phase II pilot clinical trial (secondary analysis)	60/60	tDCS	Site: anode placed over the primary motor cortex (M1), cathode placed over the contralateral supraorbital area Parameters: 2 mA current, 20 min per session, 30-s ramp-up and ramp-down current Frequency: 5 times per week, total 3 weeks (15 sessions) Mode: home-based remotely supervised intervention	Sham tDCS (only 30 s of current at the beginning and end of intervention, no sustained current)	Pain intensity: Numerical Rating Scale (NRS), WOMAC pain subscale Pain interference: WOMAC function subscale Pain catastrophizing: Pain Catastrophizing Scale (PCS)
Samuel Montero-Hernandez (38)	2023	USA	Multicenter, randomized, double-blind, sham-stimulus controlled clinical trial (RCT), also a pain neural mechanism exploration study based on previous clinical research	111	tDCS	Electrode placement: anode placed over the contralateral primary motor cortex (M1) to the affected knee, cathode placed over the ipsilateral supraorbital area (SO) to the affected knee Stimulation parameters: current intensity 2 mA, single stimulation duration 20 min, current slowly ramped up and down within 30 s at start/end Frequency: once daily (Monday to Friday weekdays) Treatment course: 3 consecutive weeks, total 15 sessions; self-administered at home	Sham tDCS stimulation, identical electrode placement, treatment frequency, course, and single duration to the active stimulation group; only 30 s of 2 mA current was given at the start and end of stimulation, no effective direct current output throughout the treatment, device kept on and electrodes connected to achieve double blinding	Clinical efficacy indicators: Numerical Rating Scale (NRS), Western Ontario and McMaster Universities Osteoarthritis Index (WOMAC), Heat Pain Threshold (HPTh), Heat Pain Tolerance (HPTo), Pressure Pain Threshold (PPT), Conditioned Pain Modulation (CPM) Neural mechanism indicators: pain-related brain functional connectivity networks detected by fNIRS (functional connectivity strength, node degree, network density, etc. of prefrontal cortex PFC, primary motor cortex M1, primary somatosensory cortex S1)
Ravi Shankar Reddy (39)	2025	Saudi Arabia	Cross-sectional Study	68	tDCS	Electrode placement: anode placed over the contralateral primary motor cortex (M1), cathode placed over the contralateral supraorbital area (SO) Stimulation parameters: current intensity 1.5–2.0 mA (average 1.95 mA), single stimulation duration 20 min Frequency/course: no standardized course, determined clinically; patients received at least 5 sessions, average 8.41 sessions	None (observational cross-sectional study of routine clinical tDCS treatment)	NPRS: Numerical Pain Rating Scale KOOS-PS: Knee injury and Osteoarthritis Outcome Score-Physical Function Short form EQ-5D-5L: EuroQol 5-Dimension 5-Level PCS: Pain Catastrophizing Scale (mediator variable)
Robert Suchting (21)	2021	USA	Randomized, double-blind, sham-controlled clinical trial (secondary data analysis)	20/20	tDCS	Site: anode (C3/C4, contralateral primary motor cortex to the affected knee), cathode (supraorbital area, contralateral to the anode) Parameters: 2 mA current, 20 min per session Frequency: once daily Treatment course: 5 consecutive days	Sham tDCS (only 30 s of weak stimulation at the beginning and end of treatment, no effective current throughout)	Core: changes in plasma Brain-Derived Neurotrophic Factor (BDNF) levels Secondary: pain scores (Numerical Rating Scale NRS, Western Ontario and McMaster Universities Osteoarthritis Index WOMAC), irisin/adiponectin/osteocalcin levels, correlation between pain improvement and BDNF changes
Juyoung Park (22)	2024	USA	Secondary analysis; original study was a double-blind, randomized, sham-stimulus controlled, phase II parallel-group clinical trial	60/60	tDCS	Electrode placement: anode placed over the primary motor cortex (M1), cathode placed over the supraorbital area (SO) Stimulation parameters: current intensity 2 mA, single stimulation duration 20 min; current slowly ramped up and down within 30 s at start/end Frequency: once daily (Monday to Friday weekdays) Treatment course: 3 consecutive weeks, total 15 sessions; self-administered at home.	Sham tDCS stimulation, identical electrode placement to the active stimulation group, but only 30 s of 2 mA current was given in the first 30 s, no current output thereafter	NRS: Numerical Rating Scale (0–100 points, assessing 24-h average pain intensity) WOMAC: Western Ontario and McMaster Universities Osteoarthritis Index (0–96 points, assessing pain and physical function) PCS: Pain Catastrophizing Scale (0–52 points, assessing pain-related catastrophic thinking)
Geraldine Martorella (40, 41)	2022	USA	Randomized, double-blind, sham-controlled, remotely supervised home-based self-administered phase II clinical trial	60/60	tDCS	Site: anode (contralateral primary motor cortex M1 to the affected knee), cathode (supraorbital area SO) Parameters: 2 mA current, 20 min per session (30-s slow ramp-up and ramp-down current) Frequency: once daily Treatment course: 3 consecutive weeks, total 15 sessions	Sham tDCS (only 30 s of electrification at the beginning, no sustained current, identical electrode placement and operation process to the active group)	Clinical: NRS pain, WOMAC osteoarthritis index, usage satisfaction, adverse events Mechanism: Quantitative Sensory Testing (QST: heat/pressure pain thresholds, Conditioned Pain Modulation CPM)
Xinmeng Zhang (23)	2025	China	Prospective randomized controlled trial (RCT)	12/11	TENS + tDCS	tDCS: anode placed over the contralateral primary motor cortex (M1, Cz point) to the affected knee, cathode placed over the ipsilateral supraorbital area; 2 mA current, 20 min per session, 3 times per week, total 6 weeks TENS: 5 cm circular electrodes attached to the medial and lateral sides of the knee; frequency 100 Hz, pulse width 100 μs, biphasic square wave, intensity 15–25 mA, 20 min per session, 3 times per week, total 6 weeks Treatment course: 6 weeks, tDCS and TENS implemented simultaneously	Sham tDCS + TENS (sham tDCS only powered on for the first 30 s then turned off, TENS parameters same as the intervention group)	Primary: VAS pain score, foot clearance height, obstacle crossing speed Secondary: hip/knee/ankle joint flexion and extension angles, vertical impulse, propulsive impulse, step height, support time
Chun-Ya Xia (24)	2025	China	Randomized, double-blind, controlled clinical trial	30/30	TENS + tDCS	tDCS: anode placed over the ipsilateral primary motor cortex (M1/C3/C4), cathode placed over the contralateral supraorbital area; 2 mA current, 2.5 s rise, 15 min maintenance, 2.5 s fall, 20 min per session TENS: 2 electrodes attached to the medial and lateral sides of the knee; 1–250 Hz scan, 60 ms pulse width, symmetric biphasic pulse, 20 min per session Treatment course: 5 times per week (Monday to Friday), total 8 weeks	Sham tDCS + TENS (sham tDCS only 5 s of ramp-up and ramp-down current, no sustained 2 mA stimulation, other operations consistent)	Pain: BPI, VAS scores Motor function: step length, step frequency, 6-min walk distance, knee range of motion, quadriceps strength Neurophysiology: electroencephalogram (EEG) *α*/*β*/*θ*/*γ* wave power changes

Yu Xu’s study included cadaver research, only clinical study data are extracted in this paper. Geraldine Martorella’s two articles are from the same study focusing on different outcomes, merged in this paper.

### 3.2 Intervention protocols and efficacy characteristics of different neuromodulation techniques

#### 3.2.1 Peripheral electrical stimulation neuromodulation

##### 3.2.1.1 Transcutaneous electrical nerve stimulation

A total of 4 studies were included, with significant heterogeneity in intervention protocols. Electrode positions were mainly divided into two categories: traditional pain areas (medial and lateral knee joint lines) and high nerve density areas (quadriceps tendon, patellar ligament, medial superior knee area); stimulation parameters mostly used 2–100 Hz, with single treatment duration of 20–60 min. In terms of efficacy, Xu et al. (10) first confirmed that placing electrodes in high nerve density areas of the knee joint resulted in significantly better efficacy than traditional pain areas, with marked improvements in pain, stiffness, and function. With technological advancement, TENS is developing toward wearability, comfort, and home use. Wearable TENS garments developed by Ullah et al. (11) and Emmanuel Maheu (12) demonstrated good home usability and pain relief effects, with better functional improvement than opioid treatment, and no serious adverse reactions (only local erythema).

##### 3.2.1.2 Neuromuscular electrical stimulation

A total of 3 studies were included, mainly targeting quadriceps atrophy, a core pathological change of KOA. Electrodes were mostly attached to motor points of the vastus medialis and vastus lateralis muscles or around the patella; current modes included AUSSIE medium-frequency alternating current, functional electrical stimulation (FES) pulse current, etc.; treatment frequency ranged from 3 times per week to 2 times per day, with treatment courses of 4–26 weeks. The multicenter extension study by Dasa et al. (13) (*n* = 62) showed that after 26 weeks of home-based NMES treatment controlled by a mobile App, patients achieved more significant pain relief compared with low-voltage sham stimulation.

##### 3.2.1.3 Peripheral nerve stimulation

Only included in the retrospective case series by Mates et al. (14), targeting 4 patients with Kellgren–Lawrence grade 4 severe KOA who received implantation of an infrapatellar saphenous nerve stimulation system. Follow-up results showed that patients achieved more than 50% pain relief and sustained functional improvement, but the sample size was extremely small, resulting in low level of evidence.

##### 3.2.1.4 Electroacupuncture (EA) and Electrical dry needling (EDN) combine the mechanical stimulation of acupuncture with the neuromodulatory effect of electrical stimulation, representing neuromodulation techniques with Chinese characteristics

*Electroacupuncture (EA)*: A total of 3 studies were included. Acupoint selection was mainly based on local knee joint acupoints, including Dubi (ST35), Neixiyan (EX-LE5), Ququan (LR8), Xiyangguan (GB33), etc., combined with meridian-based acupoint selection; stimulation parameters mostly used 2/100 Hz dense-disperse wave or continuous wave, with current intensity adjusted to induce slight needle tremor; treatment courses were 3–8 weeks, 3–6 times per week. Wang et al. (15) showed that after 8 weeks of electroacupuncture treatment, patients had greater reductions in WOMAC total score and pain score compared with the sham acupuncture group, and simultaneously increased the abundance of beneficial intestinal bacteria such as Agathobacter and Lachnoclostridium, thereby improving pain, stiffness, and functional impairment in KOA patients. Liu et al. (16) found that electroacupuncture was superior to conventional acupuncture and oral celecoxib, and could significantly reduce serum levels of pro-inflammatory factors IL-1β and TNF-*α*. The small-sample study by Jia et al. (17) first confirmed that electroacupuncture exerts anti-inflammatory effects by enhancing peripheral blood T cell receptor (TCR) diversity and reconstructing immune balance.

*Electrical dry needling (EDN)*: Only included in the multicenter RCT by Dunning et al. (18)(*n* = 586). The results showed that after the initial 6-week intensive treatment, patients who received booster treatment once every 4 weeks had sustained improvements in WOMAC total score and pain score for 30 weeks, which was significantly better than the no-booster treatment group or those who received no further treatment.

##### 3.2.1.5 Transcutaneous vagus nerve stimulation

A total of 2 studies were included, both stimulating the left cymba concha (the exclusive innervation area of the auricular branch of the vagus nerve). Stimulation parameters were 25 Hz frequency, 250 μS pulse width, with current intensity adjusted to a tolerable slight tingling sensation for patients; treatment courses ranged from a single 60-min session to 12 weeks. The 12-week RCT by Elsehrawy et al. (19) (*n* = 68) showed that tVNS could continuously reduce VAS pain score and improve pressure pain threshold (PPT), and had significant efficacy in improving knee joint injury and neuropathic pain. The single-session pilot study by Aoyagi et al. (20) confirmed that tVNS could immediately improve knee pain in patients and enhance their central analgesia and pain tolerance, with no obvious adverse reactions.

#### 3.2.2 Central neuromodulation

Transcranial direct current stimulation (tDCS): A total of 6 studies on tDCS alone were included, with relatively unified intervention protocols. Electrode positions all adopted the standard montage of placing the anode on the contralateral primary motor cortex (M1) of the affected knee and the cathode on the contralateral supraorbital area; stimulation parameters were 2 mA current intensity, 20-min single duration, with current ramping up and down within 30 s at the start and end of stimulation; treatment courses were 3 consecutive weeks, 5 times per week, total 15 times, mostly using home-based remotely supervised intervention. In terms of efficacy, most studies showed that tDCS could significantly improve pain intensity and physical function in KOA patients, and simultaneously regulate central sensitization indicators, such as increasing pressure pain threshold (PPT) and improving conditioned pain modulation (CPM) function. Suchting et al. (21) found that tDCS treatment could reduce plasma brain-derived neurotrophic factor (BDNF) levels in patients, and the reduction in BDNF was positively correlated with pain improvement. The secondary analysis by Park et al. (22) identified responder characteristics for tDCS treatment, with patients with higher body mass index (BMI) and body weight being more likely to respond to tDCS.

#### 3.2.3 Combined neuromodulation

A total of 2 studies were included, both adopting the dual-target intervention protocol of peripheral TENS combined with central tDCS. The intervention involved simultaneous implementation of local knee TENS and contralateral M1 area tDCS, with treatment courses of 6–8 weeks. Zhang et al. (23) showed that combined treatment could significantly improve gait parameters when patients stepped over obstacles, such as foot clearance height, crossing speed, and joint flexion and extension angles. Xia et al. (24) found that combined treatment could regulate the power of *α* and *β* waves in the cerebral cortex, improve pain and motor function, and was superior to TENS alone.

## 4 Discussion

### 4.1 Efficacy advantages and differential characteristics of KOA neuromodulation techniques

The results of this scoping review show that various non-surgical neuromodulation techniques have certain efficacy in KOA treatment, each with distinct advantages.

Peripheral neuromodulation techniques directly act on local nerves and muscles, with characteristics of rapid onset, simple operation, high safety, and suitability for long-term home use, making them the mainstream in clinical application. NMES directly stimulates muscles around the knee joint, mainly targeting muscle atrophy. A study by Helal B Alqurashi (25) showed that NMES had moderate to small effects on muscle size, strength, and walking performance. A systematic review by Sarah Novak (26) provided recommended NMES parameters, such as a frequency of 50–75 Hz. TENS generally has good analgesic effects with almost no side effects. Yu Wu (27) demonstrated that TENS could significantly relieve pain and improve dysfunction and walking ability in KOA patients. Electroacupuncture and electrical dry needling can simultaneously improve local inflammation, neuroimmune function, and systemic status. Yu Chen (28) found that electroacupuncture had the best efficacy among common acupuncture techniques. Studies by Zheng-tao Lv (29) and Zihang Chen (30) showed that high-intensity electroacupuncture was superior to low-intensity electroacupuncture in enhancing central analgesia, and high-intensity electroacupuncture could also improve patients’ emotional state.

Central neuromodulation techniques exert effects by regulating the brain’s pain processing network, among which tDCS is the most widely used clinically with the most abundant research. Natalia Comino-Suárez (31) indicated that tDCS has high safety and can significantly improve pain intensity and function. Rujuan Liu (32) proved that tDCS may reduce inflammation by altering the phenotypes of microglia and astrocytes and simultaneously inhibiting neural pathways. Combined neuromodulation techniques may achieve better efficacy than single techniques through synergistic effects of peripheral and central actions. A study by Rayssa Maria Do Nascimento (33) showed that tDCS combined with peripheral stimulation was superior to single stimulation in relieving pain from knee osteoarthritis and spinal cord injury. However, the number of current studies is small, and further verification is needed.

### 4.2 Current research gaps and controversies

Although the number of studies on neuromodulation for KOA has been increasing annually, there are still many gaps and controversies.

#### 4.2.1 Lack of standardization in intervention protocols

Stimulation parameters, electrode positions, treatment courses, and frequencies vary widely among different techniques, making it impossible to directly compare the efficacy of different techniques and difficult to form unified clinical guidelines. For example, TENS frequencies range from 2 Hz to 100 Hz, and there are more than 10 current modes for NMES, with optimal parameters yet to be clarified.

#### 4.2.2 Deficiencies in control design

Some studies used blank controls instead of sham stimulation controls, which may overestimate the true efficacy of neuromodulation. Meanwhile, the effectiveness of sham stimulation protocols is also controversial, as some sham stimulations may still produce weak neuromodulatory effects.

#### 4.2.3 Insufficient long-term efficacy evidence

Most studies have a follow-up period of no more than 3 months, lacking long-term efficacy and safety data beyond 6 months, making it impossible to evaluate the impact of neuromodulation on KOA disease progression.

#### 4.2.4 Incompletely elucidated mechanisms of action

Although some studies have explored mechanisms such as neuroimmunity, brain functional connectivity, and intestinal flora, the specific action pathways of different neuromodulation techniques remain unclear, making it difficult to guide individualized treatment.

#### 4.2.5 Lack of studies on special populations

Most included studies focused on middle-aged and elderly women, lacking research on men, young patients, severe KOA patients, and patients with comorbidities such as diabetes and cardiovascular diseases.

#### 4.2.6 Lack of health economic evaluation

No studies have evaluated the cost-effectiveness of neuromodulation techniques, limiting their inclusion in medical insurance policies and clinical promotion.

### 4.3 Limitations of this study

This study has the following limitations: First, limited by resources, only two English databases (PubMed and Web of Science) were included, mainly focusing on open-access literatures, which may have missed some high-quality studies. Second, the sample sizes of included studies were generally small (maximum 586 cases, minimum 3 cases), and some were self-controlled before-after trials and single-arm studies, resulting in limited levels of evidence. Third, this review focused on non-surgical neuromodulation techniques and did not include surgical neuromodulation techniques such as cryoablation and radiofrequency ablation, leading to certain limitations in the research scope. Fourth, the health economic benefits and long-term patient compliance of neuromodulation techniques were not analyzed, limiting the clinical translation value of the conclusions.

## Data Availability

The original contributions presented in the study are included in the article/supplementary material, further inquiries can be directed to the corresponding author.
