# Correction: Current status and research gaps of neuromodulation in the rehabilitation of knee osteoarthritis: a scoping review

**DOI:** 10.3389/fneur.2026.1944780

**Published:** 2026-08-04

**Authors:** Hengjia Liu, Qiang Tang

**Affiliations:** 1 Heilongjiang University of Chinese Medicine, Harbin, China; 2 The Second Affiliated Hospital of Heilongjiang University of Chinese Medicine, Harbin, China

**Keywords:** electroacupuncture, knee osteoarthritis, neuromodulation, pain, rehabilitation, transcranial direct current stimulation, transcutaneous electrical nerve stimulation

In the published article, there was an error in affiliation 1. Instead of “Graduate School, Heilongjiang University of Chinese Medicine,” it should be “Heilongjiang University of Chinese Medicine.”

The original version of this article has been updated.

